# Effect of foot–floor friction on the external moment about the body center of mass during shuffling gait: a pilot study

**DOI:** 10.1038/s41598-021-91683-5

**Published:** 2021-06-09

**Authors:** Takeshi Yamaguchi, Kei Shibata, Hiromi Wada, Hiroshi Kakehi, Kazuo Hokkirigawa

**Affiliations:** 1 Department of Finemechanics, Graduate School of Engineering, Tohoku University, 6-6-01 Aramaki-Aza-Aoba, Aoba-ku, Sendai, Miyagi 980-8579 Japan; 2 Graduate School of Biomedical Engineering, Tohoku University, Sendai, Miyagi Japan; 3 LIXIL Corporation, Tokoname, Aichi Japan

**Keywords:** Biomedical engineering, Biomechanics

## Abstract

Herein, we investigated the effect of friction between foot sole and floor on the external forward moment about the body center of mass (COM) in normal and shuffling gaits. Five young male adults walked with normal and shuffling gaits, under low- and high-friction surface conditions. The maximum external forward moment about the COM (MEFM-COM) in a normal gait appeared approximately at initial foot contact and was unaffected by floor condition. However, MEFM-COM in a shuffling gait under high-friction conditions exceeded that under low-friction conditions (*p* < 0.001). Therein, MEFM-COM increased with an increasing utilized coefficient of friction at initial foot contact; this effect was weaker during a normal gait. These findings indicate that increased friction between foot sole and floor might increase tripping risk during a shuffling gait, even in the absence of discrete physical obstacles.

## Introduction

Falling accidents are one of the factors that deter long healthy lifespans among the elderly^1^. Falls in the elderly could cause serious injuries, such as hip fractures and head trauma, deteriorating their mobility and reducing their independence^2,3^. Among the causes of long-term nursing care, bone fractures and falls were found to be the fourth-leading cause (12.1%) in 2016 in Japan^4^. In particular, in the case of femoral neck fractures, walking ability is inhibited following a long recovery period, with ~ 20% patients remaining bedridden even a year post-injury^5^.

Approximately one in two elderly falls occur within the home^6^, the most common cause being a tripping during walking^7–9^, resulting in a loss of balance in the forward direction. Age-related gait changes include reduced gait speed, step length, hip and knee extension, ankle dorsiflexion angle at heel-strike, and the clearance between the sole of the foot and floor surface, i.e., foot clearance^10–14^. Consequently, older adults could exhibit a gait similar to a shuffling gait^15,16^, thus increasing tripping^17^. Gait characteristics observed in shuffling gait such as reduced step length, reduced gait speed, and increased double support period may be attributed to stabilizing adaptations related to the fear of falling^18^.

Because tripping occurs when the foot (toes) contacts the floor or floor elevation changes, many studies have investigated the effects of floor elevation changes on falling risk^19–21^. Despite the development of barrier-free environments with no obstacles on the floor, the number of falling accidents among older adults continues to increase. Furthermore, Berg et al.^8^ reported that the two highest-ranked activities in which older fallers were engaged during the fall were walking on level and uneven surfaces (24% and 24% of all falls, respectively). Therefore, external factors beyond floor elevation changes or obstacles are deemed causes that induce tripping.

Rosen et al.^22^ reported that falls occurred because of changes in the slip resistance of the floor surface. Thus, foot–floor friction can cause trips, similar to physical objects. Menant et al.^23^ pointed out that walking barefoot or in socks over a carpeted surface might provide excessive slip resistance that could cause tripping in older adults. They also suggested that too much friction at the shoe/walking surface may be hazardous to stability among older people. However, the impact of the friction coefficient on the risk of tripping during walking has not been researched thus far. In contrast, in terms of slip-induced falls, many studies have investigated the effects of the friction coefficient on the risk of slip and slip-induced falls, resulting in proposals for a minimum friction coefficient at the shoe–floor interface to prevent slipping^24–31^.

Tripping is an action in which the body is thrust in the forward walking direction. When friction force is applied backward at initial foot contact, the force impulse rotates the body in the forward direction^32^. Thus, the large friction at the foot–floor contact will increase the forward moment about the whole-body center of mass (COM), which increases the forward angular momentum of the entire body^19^. Because a shuffling gait requires the foot to slide across the floor, we hypothesized that the friction coefficient between the foot sole and the floor would have more impact on the external moment about the body COM during a shuffling gait than during a normal gait. In particular, we hypothesized that on a high-friction floor, a shuffling gait would suffer from a larger external moment about the COM than a normal gait. Therefore, this study investigated the effect of the change in friction coefficient on the forward COM moment during the shuffling and normal gait. As a pilot study, we tested the above hypothesis with young adult male participants.

## Methods

### Participants

This study included five healthy young adult males. The age, height, and body mass of the participants were 22.8 ± 0.43 years, 1.68 ± 0.05 m, and 61.3 ± 5.4 kg (mean ± standard deviation), respectively. All methods/experiments were performed in accordance with relevant guidelines and regulations. The participants were informed of the protocol, and informed consent was obtained from each participant before the experiment. The protocol was approved by the Institutional Review Board of Tohoku University.

### Experimental procedure

The experimental setup used in this experiment comprises a walkway (length of 5 m), two force plates (MG-2060, ANIMA Corporation), eight infrared cameras (Locus 3D MA-8000, ANIMA Corporation), an A/D converter, and a personal computer. Three-dimensional motion analysis software (MA-8000, ANIMA Corporation) and infrared cameras were used to examine subject motion using 16 infrared-reflective markers placed bilaterally over the acromion, lateral epicondyle of the humerus, stylion ulnare, anterior superior iliac spine, trochanter, lateral epicondyle of the femur, sphyrion fibulare, and fifth metatarsal heads. Two force plates were installed on the walkway to measure ground reaction forces (GRF), with a total measurement area of 0.6 × 1.2 m. The *x*-axis was set to the right side relative to the walking direction, the *y*-axis was pointing in the forward walking direction, and the *z*-axis in the vertical direction normal to the walkway. The sampling frequency for kinematics and kinetics data was 500 Hz.

Participants wore athletic undergarments and sportswear that kept close contact with the skin to avoid displacement between the marker and the desired location during body motion. Menz et al.^33^ reported that those who fell indoors were more likely to walk barefoot or wear socks inside the home; thus, we asked each participant to wear the same type of sock, with the following material composition: 36.7% cotton, 32.8% polyester; 23.2% acrylic, 4.8% nylon (polyamide), and 2.5% polyurethane. This also established uniformity and established sole focus on the change in floor material and the friction coefficient between the foot sole and the floor.

Participants were instructed to walk in two different walking styles, i.e., the normal and shuffling gaits. In the normal gait, participants were asked to walk naturally with a step length of 40% of the body height and cadence of 110 steps/min^34^. In the shuffling gait, they were instructed to walk keeping as much contact between the foot sole and the floor surface as possible, with a step length of 20% of the body height and cadence of 100 steps/min, which were set based on the reported examples of walking found among the elderly^35^. The step length was outlined by tape markers placed alongside the walking path and the cadence was specified using a metronome.

**Figure 1:**
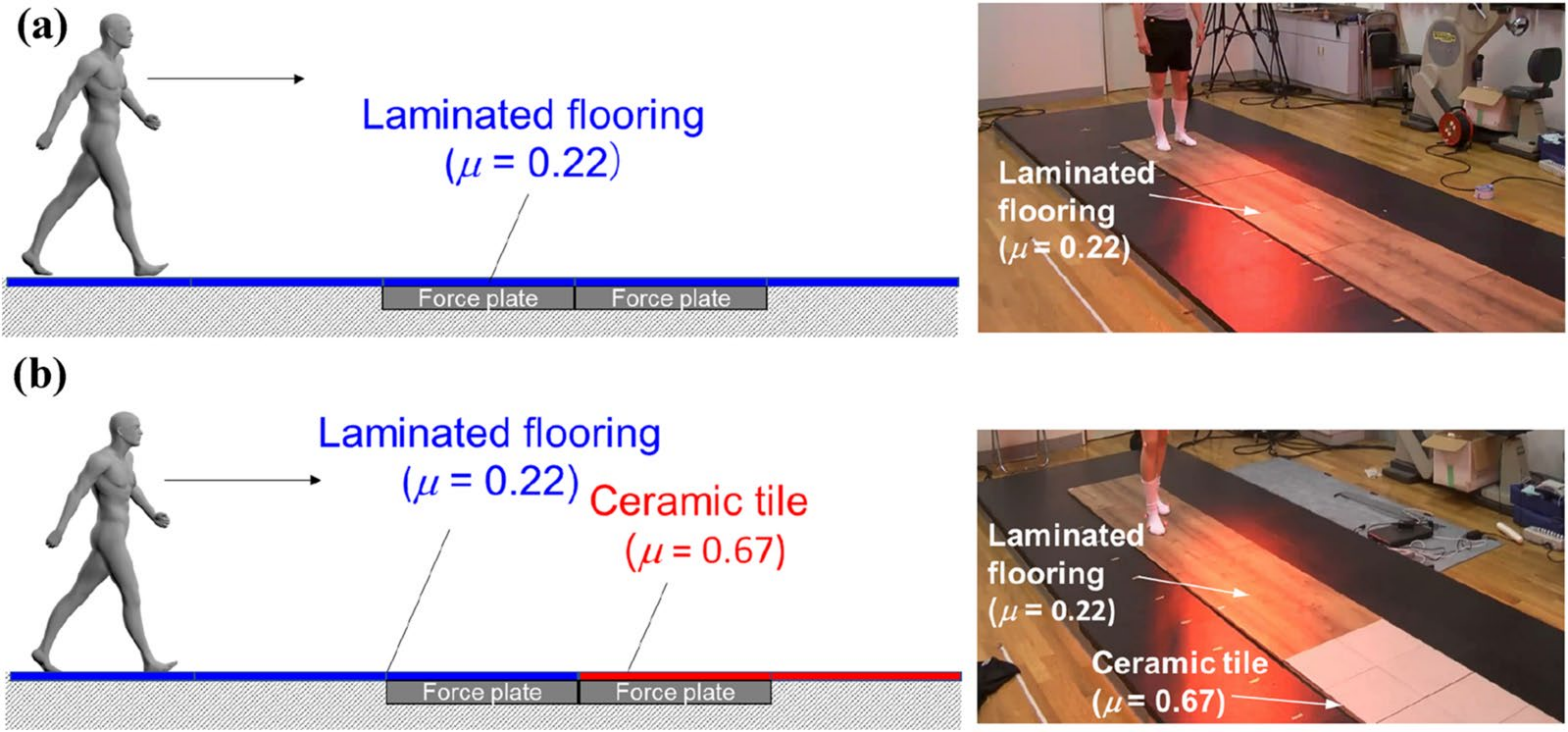
Floor combinations. (**a**) Low-friction and (**b**) high-friction conditions were used in this experiment. Low-friction conditions used only laminated wood flooring, whereas high-friction conditions included a transition from laminated wood flooring to the ceramic tile in the forward walking direction. The flooring and tiles for high-friction conditions were placed alongside the boundaries between the two force plates; the laminated wood flooring on the first force plate, and the ceramic tile on the second, arranged in the forward walking direction.

**Figure 2:**
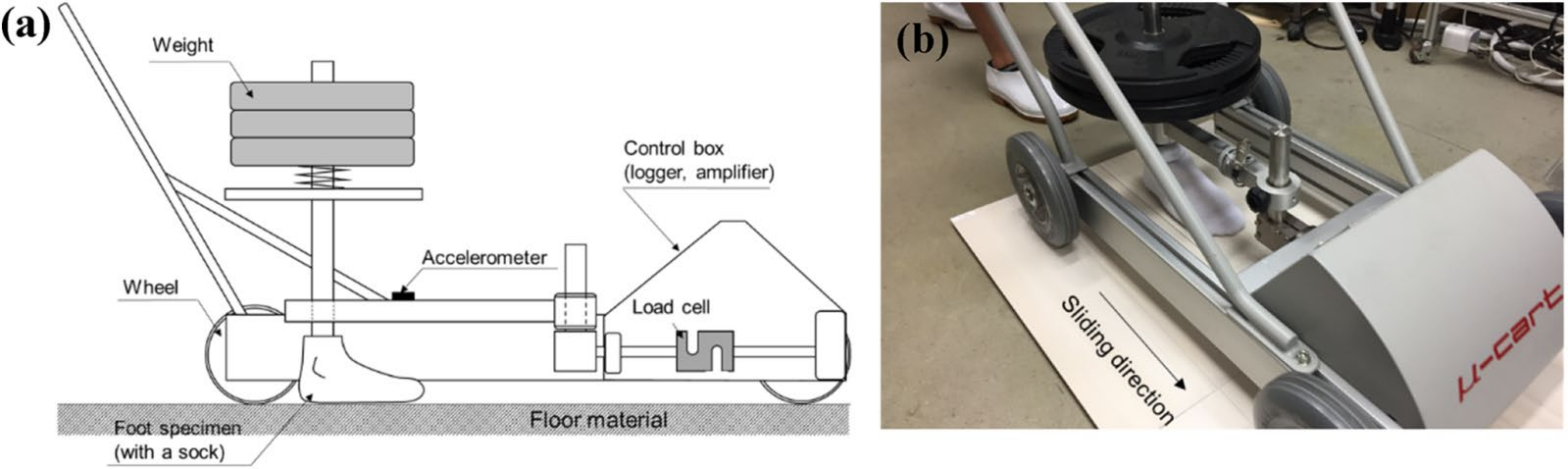
Cart-type friction measurement device. (**a**) Schematic of the cross-sectional view of the mechanical system configuration and (**b**) photo of friction measurement.

Figure 1 illustrates the combination of floor materials used in this experiment. Floor materials with high and low coefficients of friction relative to socks were selected beforehand through friction testing. The socks used in the walking experiments were placed on a foot specimen made of silicone, and the coefficient of friction relative to the floor material was measured using a cart-type friction measurement device^36,37^ (μ-cart, Trinity Lab Corporation) as shown in Fig. 2. The cart-type friction measurement device was pushed by the experimenter on the floor surface, and the friction coefficient at various sliding velocities was measured. The measurement principle of the friction coefficient between foot specimen and floor specimen is described elsewhere ^36,37^. The vertical load of the testing device was set at 217 N. We performed friction tests for 20 different floor materials. The test was performed five times per floor material. Among these materials, we selected the two exhibiting the universally highest (a ceramic tile) and lowest friction coefficients (a laminate wood flooring material), such that the difference in friction coefficient between two successive flooring samples in the gait trial would be maximized. At an average sliding speed of 0.2 m/s, the dynamic friction coefficient between socks and the laminate wood flooring was measured at a mean value (± standard deviation (SD)) of 0.22 (± 0.01), and 0.67 (± 0.04) for the ceramic tile. Using these two flooring materials, low friction and high friction conditions were applied in this experiment. The low-friction condition used only laminated wood flooring, whereas the high-friction condition used a transition from laminated wood flooring to the ceramic tile in the forward walking direction.

To clarify the effects from the transition in flooring material, the flooring and tiles for the high-friction condition were placed in line with the boundaries between the two force plates; the laminated wood flooring on the first force plate and ceramic tile on the second, arranged in the forward walking direction. The floor material was fixed to the walkway and force plates with double-sided tape to avoid displacement during walking. Furthermore, a space of about 1–2 mm was set between the laminated wood and ceramic tile to avoid contact or interference. To ensure uniform height of the floor material placed along the walkway, a wood spacer was used to elevate the thinner ceramic tile to eliminate the physical level differences.

Each participant started walking about 3 m before the force plate. The initial walking step and contact foot with the second force plate (high friction floor surface for high-friction condition) made with the left or right side, was unspecified and left up to the participant’s personal preference. Before the gait trials, participants could walk alongside the walkway, where no material was installed, several times according to the specified stride and walking rate, to get accustomed to the required foot placement. Each participant walked five times under the same conditions, 20 times in total (2 walking methods × 2 floor combinations × 5 repetitions). The order of each block of trials (normal gait-low friction, normal gait-high friction, shuffling gait-low friction, shuffling gait-high friction) was randomized. The participants could redo their walking trial if their foot landed on the boundary between the two force plates or if their walking rate or step length significantly deviated from the specified conditions.

### Data analysis

Foot clearance and the foot–floor angle were investigated to examine if those values differed between the normal and shuffling gaits. Foot clearance was defined as the minimum distance between the foot sole and the floor in each swing and stance phase. In this context, the foot clearance during each stance phase should be zero. Herein, we determined foot clearance using the *z*-coordinates of the ankle and metatarsal markers. The *z*-coordinate of each marker during standing still (*z*_ank_0_ and z_meta_0_) was subtracted from the *z*-coordinate of each marker during walking (*z*_ank_ and z_meta_), which was used as the height of the sole beneath the ankle and metatarsal. The lower value of the two heights (*z*_ank_ − *z*_ank_0_ and *z*_meta_ − *z*_meta_0_) in each swing and stance phase was selected as the foot clearance. The angle of the line connecting the ankle and metatarsal markers with the horizontal line during standing still was subtracted from one during walking. This angle difference was determined as the foot–floor angle in each swing and stance phase during walking.

Lower limb kinematics between normal and shuffling gaits were compared. Hip, knee, and ankle joint angles in the sagittal plane were calculated. The hip joint angle was defined as the angle between trunk (acromion–trochanter) and thigh (trochanter–lateral epicondyle of femur) segments; the knee joint angle was defined as the angle between thigh (trochanter–lateral epicondyle of femur) and shank (lateral epicondyle of femur–sphyrion fibulare) segments; and the ankle joint angle was defined as the angle between the vector perpendicular to lateral epicondyle of femur–sphyrion fibulare and foot segment (sphyrion fibulare–fifth metatarsal heads)^38^.

**Figure 3:**
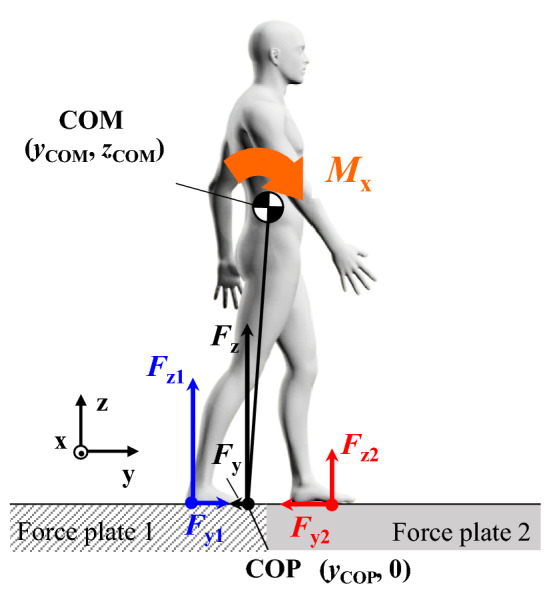
Schematic of the measured and calculated kinetic and kinematic variables in the sagittal plane. *F*_*y*1_ and *F*_*y*2_ are the anteroposterior GRF of the first and second force plates, respectively. *F*_*z*1_ and *F*_*z*2_ are the vertical GRF of the first and second force plates, respectively. *y*_COP_ is the global COP in the anteroposterior direction. *y*_COM_ and *z*_COM_ are the position of whole-body COM in the anteroposterior and vertical directions, respectively. *M*_*x*_ is the external moment about COM in the sagittal plane.

The utilized coefficient of friction at foot–floor contact and external moment about body COM in the sagittal plane were calculated. As shown in Fig. 3, the anteroposterior GRF of the first force plate (*F*_*y*1_) and the second force plate (*F*_*y*2_) and the vertical GRF of the first force plate (*F*_*z*1_) and the second force plate (*F*_*z*2_) were collected. The COP of each individual force plate in the local coordinate system (*Y*_COP1_ and *Y*_COP2_) was computed using MA-8000 software according to the following equations

1 $${Y}_{COPi}=\frac{{M}_{Xi}}{{F}_{Zi}} \quad (i=\mathrm{1,2})$$

2 $${M}_{Xi}=\left(-{F}_{Zi,1}-{F}_{Zi,2}+{F}_{Zi,3}+{F}_{Zi,4}\right)\frac{\sqrt{2}}{2}r  \quad(i=\mathrm{1,2})$$

3 $${F}_{zi}=\left({F}_{Zi,1}+{F}_{Zi,2}+{F}_{Zi,3}+{F}_{Zi,4}\right)  \quad (i=\mathrm{1,2})$$

here, ${M}_{Xi}$ represents the moment about the *X*-axis acting on the force plate, and *i* represents the index number of the force plate. *F*_Z*i*,1_, *F*_Z*i*,2_, *F*_Z*i*,3_, and *F*_Z*i*,4_ represent the vertical forces detected by force sensors located in the four corners of the force plate. *r* represents the distance from the center of the force plate to each force sensor. The global COP in the anteroposterior direction *y*_COP_ was calculated using the local COP locations in the global coordinate system (*y*_COP1_ and *y*_COP2_, respectively) as

4 $${y}_{\mathrm{COP}}={y}_{\mathrm{COP}1}+\frac{{F}_{\mathrm{z}2}}{{F}_{\mathrm{z}1}+{F}_{\mathrm{z}2}}\left({y}_{\mathrm{COP}2}-{y}_{\mathrm{COP}1}\right).$$

The position of whole-body COM in the sagittal plane (*y*_COM_, *z*_COM_) was estimated using a seven-segment rigid link model (trunk, bilateral feet, shanks, and thighs) involving kinematic data using motion analysis software (MA8000). The COM of each segment was computed from each marker location and body-segment inertia parameter^39^. The COM of the whole body was computed as

5 $${y}_{COM}=\frac{\sum_{j=1}^{7}{m}_{j}{y}_{COMj}}{M},$$

6 $${z}_{COM}=\frac{\sum_{j=1}^{7}{m}_{j}{z}_{COMj}}{M},$$

where *m*_*j*_ represents the mass of the *j*th body segment, *y*_COM*j*_ and *z*_COM*j*_ represent the coordinates of the COM location of the *j*th body segment, and *M* represents the whole-body mass.

Second-order, zero-lag, Butterworth low-pass filtering with 50- and 10-Hz cutoff frequencies were performed on the GRF and kinematic data, respectively, using MATLAB (Mathworks, Natick, MA, USA). MATLAB was also used for subsequent analyses. The utilized coefficients of friction in the sagittal plane for the first and second force plates *ϕ*_1_ and *ϕ*_2_, respectively, were defined as the ratio between the anteroposterior and the vertical GRF (*F*_*y*1_/*F*_*z*1_ and *F*_*y*2_/*F*_*z*2_), respectively. The external moment around the whole-body COM in the sagittal plane was calculated as^40–42^

7 $${M}_{x}={(F}_{z1}+{F}_{z2})\left({y}_{\mathrm{COM}}-{y}_{\mathrm{COP}}\right)-{(F}_{y1}+{F}_{y2}){\mathrm{z}}_{\mathrm{COM}}.$$

**Figure 4:**
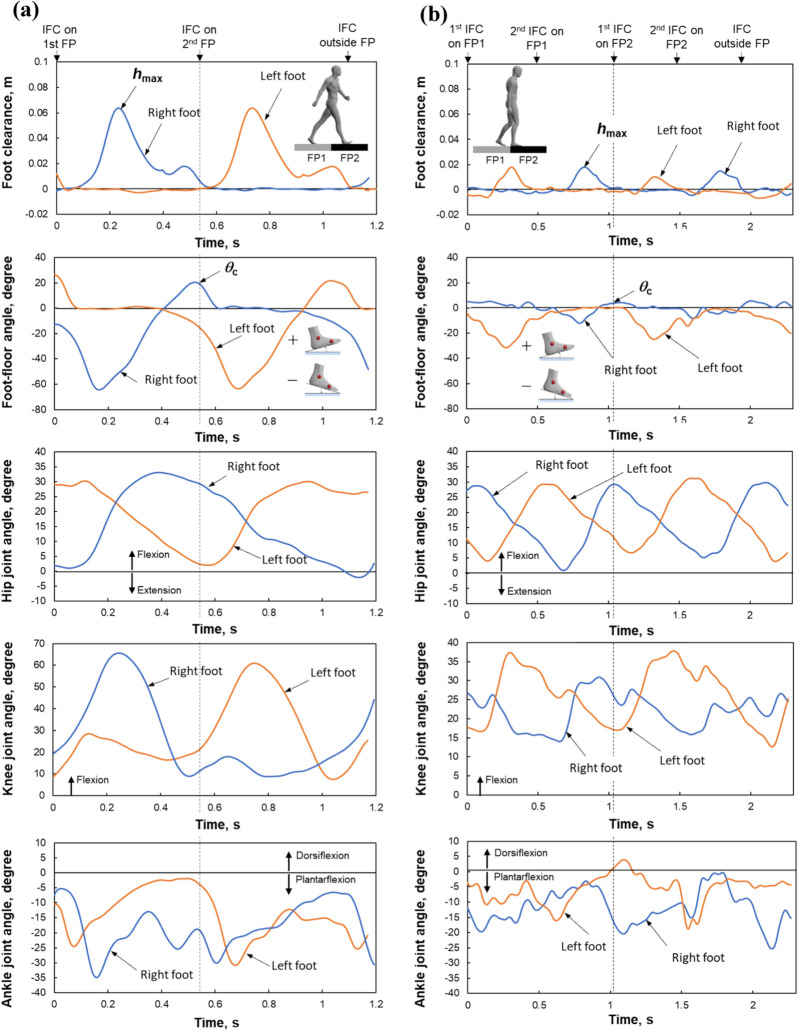
Example of the temporal change for a single subject in the foot clearance, foot–floor angle, hip joint angle, knee joint angle, and ankle joint angle during the normal (**a**) and shuffling gaits (**b**) under the low friction condition. *h*_max_ is the maximum value of the foot clearance before the foot contact on the second force plate, corresponding to the local maxima of the toe clearance. *θ*_c_ is the foot–floor angle at initial foot contact (IFC) on the second force plate. IFC timing for each step except the first step on the first and second force plates (FPs) were indicated with arrows.

**Figure 5:**
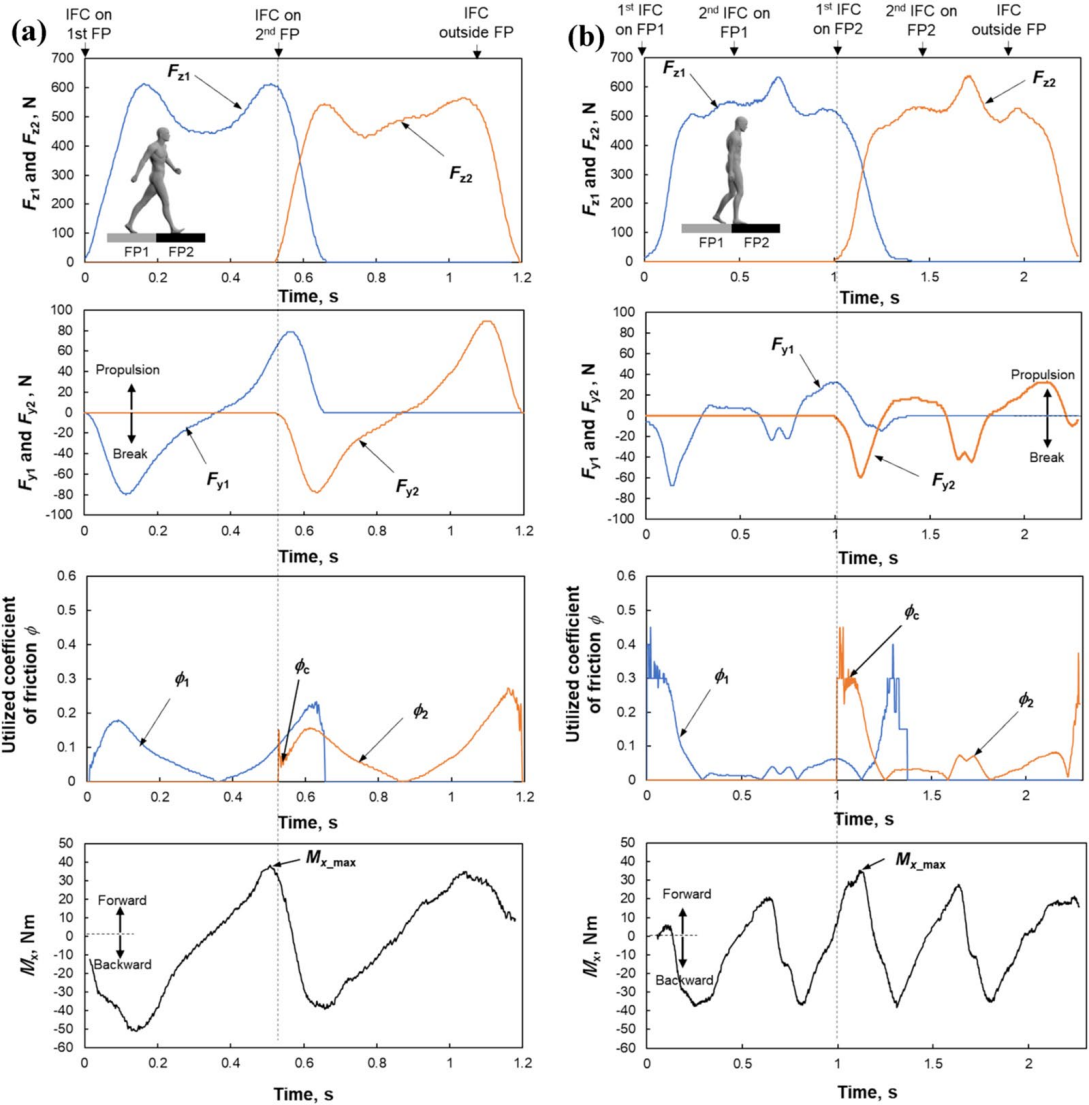
Example of the temporal change for a single subject in the vertical GRFs (*Fz*_1_ and *Fz*_2_), anterior–posterior GRFs (*F*_*y*1_ and *F*_*y*2_), the utilized coefficient of friction (*ϕ*_1_ and *ϕ*_2_), and the external moment about COM (*M*_x_) during the normal (**a**) and shuffling gait (**b**) under low-friction conditions. *M*_x_max_ is the maximum external forward moment about COM. *ϕ*_c_ is the utilized coefficient of friction at the timing when the *F*_z2_ was > 50 N. Initial foot contact (IFC) timing for each step except the first step on the first and second force plates (FPs) were indicated with arrows.

Figure 4 shows typical temporal patterns in the kinematic data, including foot clearance, foot–floor angle, hip joint angle, knee joint angle, and ankle angle for both feet, from initial foot-contact on the first force plate to foot-off from the second force plate during normal (Fig. 4a) and shuffling gaits (Fig. 4b) under low-friction conditions. Figure 5 shows the temporal change in the kinetic data including the vertical GRFs (*F*_*z*1_ and *F*_*z*2_), the anterior–posterior GRFs (*F*_*y*1_ and *F*_*y*2_), the utilized coefficient of friction (*ϕ*_1_ and *ϕ*_2_), and the external moment about COM (*M*_x_) during the normal (Fig. 5a) and shuffling gaits (Fig. 5b) under the same floor condition. The duration for *F*_*z*1_ and *F*_*z*2_ during the shuffling gait is longer than that during the normal gait because two steps were taken on each force plate during the shuffling gait, whereas a single step was taken on each force plate during the normal gait. The maximum value of the foot clearance *h*_max_ before foot contact on the second force plate, corresponding to the local maxima of the toe clearance^43^, was used to compare the normal and shuffling gaits (Fig. 4). The foot contact angle *θ*_c_ was defined as the foot–floor angle at initial foot contact on the second force plate (Fig. 4). The joint angle for each joint at the initial foot contact on the first force plate, maximum and minimum joint angles during the whole gait cycle, and range of motion (ROM) of joint angle during the whole gait cycle for each lower limb joint were used to compare the normal and shuffling gait. The maximum external forward moment about COM (*M*_x_max_) was observed approximately at the initial foot contact for the normal gait but ~ 100 ms after the initial foot contact for the shuffling gait. We collected the *M*_x_max_ value and utilized coefficient of friction *ϕ*_c_ at the instant when *F*_z2_ surpassed 50 N.

### Statistical analysis

Statistical analyses were performed using SPSS Statistics for Windows, Version 19.0 (IBM, Armonk, NY, USA). We performed a paired *t* test to investigate whether the gait type affected the kinematic data including *h*_max_, *θ*_c_, the joint angle for each joint at the initial foot contact on the first force plate, maximum and minimum joint angles during the whole gait cycle, and ROM of joint angle during the whole gait cycle for each lower limb joint. We also performed a two-way repeated-measures ANOVA to investigate whether the gait type and floor condition affected *M*_*x_*max_, and *ϕ*_c_. A post-hoc paired *t* test with a Bonferroni correction was used to determine specific significant differences between types of gait and floor conditions. We also reported effect size in terms of *η*^2^_p_ for two-way ANOVA and Cohen’s *d* for *t*-tests. *p *values < 0.05 indicated statistical significance.

## Results

### Foot clearance, foot contact angle, and lower limb joint angle

Mean and SD values of kinematic parameters are listed in Table 1. *h*_max_ and *θ*_c_ during shuffling gait were significantly lower than those during normal gait (*p* < 0.01, Cohen’s *d* = 3.34 for *h*_max_; *p* < 0.01, Cohen’s *d* = 3.95 for *θ*_c_). Among lower limb joint angle parameters, shuffling gait exhibited lower ROM of hip joint angle (*p* < 0.01, Cohen’s *d* = 2.10), lower ROM of knee joint angle (*p* < 0.05, Cohen’s *d* = 1.45), and larger knee joint angle at initial contact (*p* < 0.05, Cohen’s *d* = 1.91) than the normal gait. The other lower limb joint parameters were not significantly affected by the type of gait (*p* > 0.05).

**Table 1:** Mean and SD values for maximum foot clearance, foot–floor angle at initial foot contact, and lower-limb joint kinematics parameters.

Parameters	Normal gait		Shuffling gait		p value	Cohen’s *d*
	Mean	SD	Mean	SD		
Maximum foot clearance *h*_max_ (m)	0.09	0.01	0.04	0.02	< 0.01**	3.34
Foot contact angle *θ*_c_ (°)	22.54	2.93	− 3.95	4.84	< 0.01**	3.95
**Hip joint angle (°)**						
Initial contact	27.53	3.31	25.90	3.46	0.502	0.33
Max	31.06	2.77	28.81	3.32	0.333	0.49
Min	− 3.94	6.22	2.84	2.58	0.094	0.98
ROM	35.00	4.16	25.97	1.13	< 0.01**	2.10
**Knee joint angle (°)**						
Initial contact	14.18	6.23	31.81	4.27	0.013*	1.91
Max	69.38	4.10	53.31	12.63	0.056	1.19
Min	− 3.94	6.22	2.84	2.58	0.094	0.98
ROM	57.44	5.83	35.62	12.18	0.031*	1.45
**Ankle joint angle (°)**						
Initial contact	− 13.65	4.66	− 11.22	4.63	0.546	0.29
Max	0.35	3.94	3.69	2.86	0.253	0.60
Min	− 31.66	3.37	− 23.98	7.58	0.125	0.87
ROM	32.01	4.34	27.67	6.30	0.135	0.84

*p < 0.05.

**p < 0.01.

### Maximum forward external moment about COM and utilized coefficient of friction at foot contact

**Figure 6:**
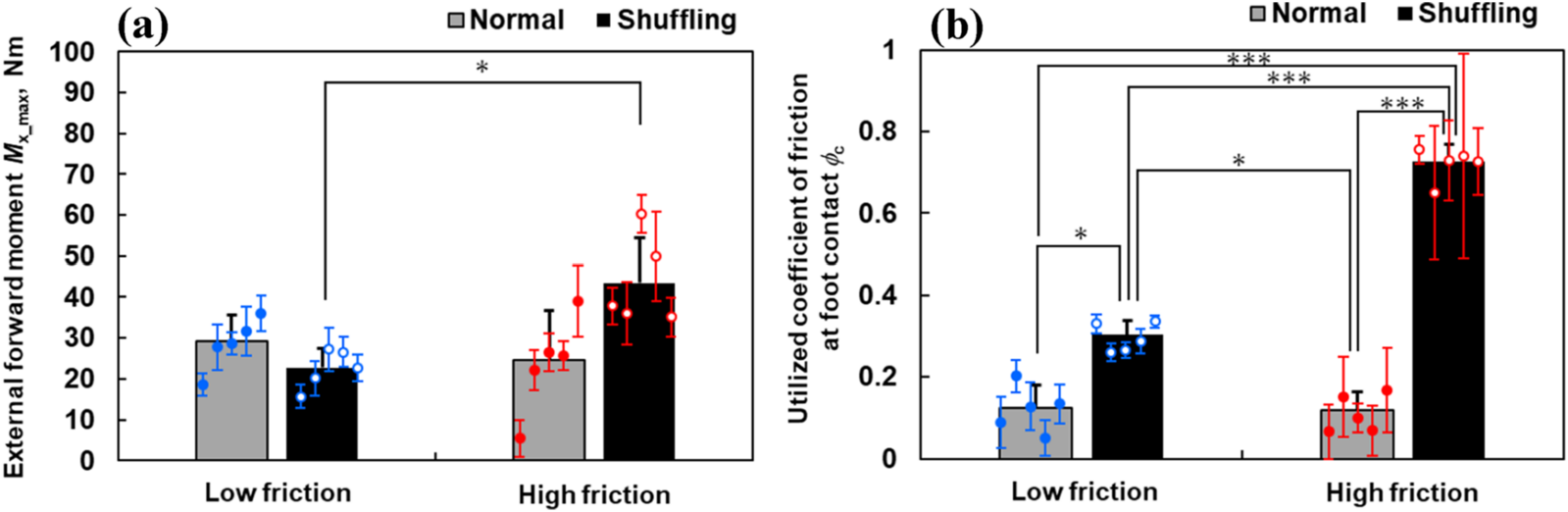
Comparison of mean and SD values of (**a**) *M*_*x*_*_*_max_ and (**b**) *ϕ*_c_ during normal and shuffling gait trials under the low- and high-friction conditions. Error bars around each plotted point represent SD values around each subject’s mean. **p < 0.01, ***p < 0.001.

Figure 6a,b show the mean value of *M*_*x_*max_ and *ϕ*_c_ for low-friction and high-friction conditions during normal and shuffling gait trials, respectively. Error bars around each plotted point represent SD values around each subject’s mean. The two-way repeated-measures ANOVA indicated that *M*_*x_*max_ was not significantly affected by gait type [*F*(1, 4) = 2.399, *p* = 0.237, *η*^2^_p_ = 0.327] but was significantly affected by floor condition [*F*(1, 4) = 17.424, *p* < 0.05, *η*^2^_p_ = 0.813] and by gait type–floor condition interaction [*F*(1, 4) = 26.629, *p* < 0.01, *η*^2^_p_ = 0869]. A post-hoc analysis revealed that *M*_*x_*max_ for shuffling gait under the high-friction floor condition was significantly larger than that under the low-friction condition (*p* < 0.05, Cohen’s *d* = 2.619) (Fig. 6a). *ϕ*_c_ was significantly affected by gait type [*F*(1, 4) = 136.442, *p* < 0.001, *η*^2^_p_ = 0.972], floor condition [*F*(1, 4) = 350.855, *p* < 0.001, *η*^2^_p_ = 0.989], and gait type–floor condition interaction [*F*(1, 4) = 379.046, *p* < 0.001, *η*^2^_p_ = 0.990]. A post-hoc analysis (Fig. 6b) demonstrated that *ϕ*_c_ during the shuffling gait was significantly larger than that during the normal gait under both low-friction (*p* < 0.05, Cohen’s *d* = 6.314 for normal gait) and high-friction conditions (*p* < 0.001, Cohen’s *d* = 7.532 for normal gait). Furthermore, *ϕ*_c_ under high-friction conditions was significantly larger than that under low-friction conditions during the shuffling gait trial (*p* < 0.01, Cohen’s *d* = 12.150) (Fig. 6b).

## Discussion

Comparison of lower limb joint angle parameters between normal and shuffling gaits indicated that the ROM of the hip and knee joint angle for the shuffling gait was lower than that for the normal gait while the ROM of ankle joint angle was not significantly affected by the type of gait. Knee joint angle at initial foot contact for the shuffling gait was significantly larger than that for the normal gait. The reduction in the ROM of hip and knee joint angle and the increase in the knee joint angle at initial foot contact are also observed in older adults with Parkinson’s disease (PD) who are likely to have a shuffling gait^44,45^. The results also indicated that the hip and knee extension tended to decrease in shuffling gait compared to normal gait (*p* < 0.1, Cohen’s *d* = 0.98), which is observed in elderly fallers^11,46^. The results of the maximum foot clearance *h*_max_ and the foot contact angle *θ*_c_ demonstrated that the values when walking with a shuffling gait were significantly reduced compared to those when walking with a normal gait. The reduced foot clearance in a shuffling gait was possibly due to the reduced maximum knee flexion angle. As shown in Table 1, the mean value of *θ*_c_ was negative (− 3.95 ± 4.84°) during the shuffling gait, indicating that the foot contacted with flat foot or a forefoot during the shuffling gait, whereas during normal gait, the foot contacted initially with a heel because the mean value of *θ*_c_ was positive (22.54 ± 2.93°). This was possibly due to the increased knee flexion at the initial foot contact in a shuffling gait. These results may indicate that the kinematics of shuffling gait by young adult participant in this study might well simulate the shuffling gait observed in older adults.

Herr and Popovic^40^ calculated the moment about COM in the sagittal plane during normal gait using the same equation as that used herein. Their results indicated that the peak forward moment appeared just before 50% gait cycle (approximately at the initial contact timing of leading foot) and the peak backward moment appeared at around 60% gait cycle (approximately the loading phase of the leading foot), which is identical with our results of the moment about COM during normal gait as shown in Fig. 5. Our results showed that the external forward moment about COM *M*_x_max_ during normal gait was not different between the low-friction and high-friction conditions, whereas that during the shuffling gait was significantly affected by the floor conditions; *M*_x_max_ under the high-friction condition was larger than that under the low-friction conditions (Fig. 6a). We also found that the utilized coefficient of friction *ϕ*_c_ did not differ between low-friction and high-friction conditions when walking with a normal gait. However, *ϕ*_c_ during the shuffling gait was significantly larger than that during the normal gait under both low-friction and high-friction conditions, and *ϕ*_c_ during the shuffling gait under high-friction conditions was larger than that under low-friction conditions (Fig. 6b). These results show that *M*_x_max_ and *ϕ*_c_ during normal gait are unaffected by the change of floor friction. However, those during the shuffling gait are significantly affected by the change in floor friction and the increased friction at foot sole and floor contact increases the external forward moment about COM at initial foot contact during the shuffling gait, which may increase the tripping risk, supporting our hypothesis.

The floor friction had little effects on the utilized coefficient of friction during the normal gait with the selected step length and walking velocity in this study because the utilized coefficient of friction did not reach the static friction coefficient, resulting in no slipping at foot contact^25^. However, when walking with a shuffling gait, the utilized coefficient of friction reached the static friction coefficient between foot and the floor on the second force plate at initial foot contact; therefore, the contacting foot slid against the floor surface. Therefore, the *ϕ*_c_ during the shuffling gait (corresponding to the friction coefficient) was larger than that during the normal gait and was affected by the difference in the floor friction. This floor friction difference results in the difference in the horizontal GRF at initial foot contact on the second force plate (*F*_*y*2_). As shown in Fig. 5b, the *F*_*y*2_ peak occurs earlier and the *F*_z2_ was lower at the instance of *M*_x_max_ in a shuffling gait. We confirmed the positive correlation between *M*_x_max_ and *F*_*y*2_ at the instance of *M*_x_max_ for all of the trials (Pearson correlation coefficient *r* = 0.65, *p* < 0.005). Thus, the increased *M*_x_max_ during the shuffling gait could be because of the increased *F*_*y*2_ on the high friction floor, resulting in an increased horizontal GRF (*F*_*y*1_ + *F*_*y*2_) in Eq. (7), despite the reduced moment arm for vertical GRF, i.e., *y*_COM_–*y*_COP_ associated with the shorter step length. As shown in Fig. 6a, *M*_x_max_ during the normal gait and shuffling gait under low-friction conditions was not significantly different although *ϕ*_c_ during the shuffling gait was significantly larger than that during the normal gait. This could be because the small *y*_COM_–*y*_COP_ values associated with the shorter step length during shuffling gait canceled the effect of the increased *F*_*y*2_.

Because the forward external moment about COM is larger in a shuffling gait than in normal gait under high friction condition, a recovery step with longer step length may be needed in shuffling gait to regulate (reduce) the whole-body angular momentum. We calculated the step length of the second step on the second force plate as a recovery step length in the shuffling gait trials. The recovery step lengths for low- and high-friction conditions were found to be nearly identical at 0.354 ± 0.056 and 0.357 ± 0.042 m, respectively (paired *t* test, *p* > 0.05). We also calculated step width, and step direction (horizontal angle of the recovery step) and found no significant differences in these variables between low- and high-friction conditions (paired *t* test, *p* > 0.05). Although our results indicated no significant differences in recover step characteristics, the more increased friction coefficient of the second floor may increase forward external moment and affect the recovery step characteristics. Studies in the literature indicate that the first recovery step after forward balance loss is shorter in older adults than in young adults ^47^. Because we used young adult male subjects in this study, the effect of the friction-induced forward COM moment on recovery steps in older adult subjects requires further research.

Our results could provide new insight into the footwear and floor design for older adults and PD patients who are likely to walk with a shuffling gait^48^. Excessive high friction shoes and floors might be inappropriate for these individuals regarding the prevention of trip-induced falls; thus, we might have an optimal range of friction coefficient at shoe and floor contact for those who walk with a shuffling gait to reduce the risk of both slip- and trip-induced falls. We asked participants to walk with a shuffling gait on whole high friction floor using ceramic tile floors; however, it was difficult for the participants to keep walking with a shuffling gait because of the high friction. Thus, a floor with an appropriately low friction coefficient is necessary for individuals who walk with shuffling. Further research is needed to explore the optimal friction range required for the contact between foot or shoe sole and floor via gait trials using multiple floor combinations with different friction levels. Our pilot study also revealed that floor friction changes from low value to high value could increase the risk of tripping; therefore, friction barrier-free floor surfaces might be required for those who are likely to walk with a shuffling gait (older adults and PD patients).

Only two floor combinations (low-friction and high-friction conditions) were used herein, which limits our study. According to Menant et al.^23^, walking barefoot or in socks over a carpeted surface might provide excessive slip resistance that could lead to older adults tripping. Therefore, further investigation is needed using a carpeted floor and other types of floors. In this study, there were no falls in the trials; therefore, it is challenging to assess the risk of trip-induced falls in this study. In the future, we must also investigate how the friction between the foot sole and floor influences the risk of trip-induced falls during the shuffling gait and whether our results apply to elderly people with or without impaired health and mobility.

## Conclusion

This pilot study is the first attempt to investigate the effect of friction change between the foot sole and the floor on the external forward moment about COM during normal gait and shuffling gait. In conclusion, during the shuffling gait, the maximum external forward moment about COM applied at the initial foot contact was affected by the foot sole–floor friction. The external forward moment increased with the increasing utilized coefficient of friction between the foot sole and floor at the initial foot contact during shuffling gait. In contrast, the external forward moment about COM at the initial foot contact was unaffected by the foot sole–floor friction during normal gait. The results demonstrated that the increased friction between the foot sole and floor could affect postural balance, leading to tripping when walking with a shuffling gait. The results might provide new insights into the design of shoes and floors for those walking with a shuffling gait.

## Data Availability

The data that support the findings of this study are available from the corresponding author upon reasonable request.
